# The differential distributions of ASPM isoforms and their roles in Wnt signaling, cell cycle progression, and pancreatic cancer prognosis

**DOI:** 10.1002/path.5341

**Published:** 2019-10-23

**Authors:** Chung‐Chi Hsu, Wen‐Ying Liao, Tze‐Sian Chan, Wei‐Yu Chen, Chung‐Ta Lee, Yan‐Shen Shan, Po‐Jui Huang, Ya‐Chin Hou, Chi‐Rong Li, Kelvin K Tsai

**Affiliations:** ^1^ Laboratory of Advanced Molecular Therapeutics Wan Fang Hospital, Taipei Medical University Taipei Taiwan; ^2^ Graduate Institute of Clinical Medicine College of Medicine, Taipei Medical University Taipei Taiwan; ^3^ Integrative Therapy Center for Gastroenterologic Cancers Wan Fang Hospital, Taipei Medical University Taipei Taiwan; ^4^ Division of Gastroenterology, Department of Internal Medicine Wan Fang Hospital, Taipei Medical University Taipei Taiwan; ^5^ School of Medicine, College of Medicine Taipei Medical University Taipei Taiwan; ^6^ Department of Pathology Wan Fang Hospital, Taipei Medical University Taipei Taiwan; ^7^ Department of Pathology National Cheng‐Kung University Hospital Tainan Taiwan; ^8^ Department of Surgery National Cheng‐Kung University Hospital Tainan Taiwan; ^9^ Department of Teaching and Research Taichung Hospital, Ministry of Health and Welfare Taichung Taiwan; ^10^ National Institute of Cancer Research National Health Research Institutes (NHRIs) Zhunan Taiwan

**Keywords:** ASPM, isoform, pancreatic cancer, Wnt, stemness, cell cycle

## Abstract

Pancreatic ductal adenocarcinoma (PDAC) is a highly aggressive and treatment‐resistant malignancy. The lack of pathway‐informed biomarkers hampers the development of rational diagnostics or therapies. Recently, the protein abnormal spindle‐like microcephaly‐associated (ASPM) was identified as a novel Wnt and stemness regulator in PDAC, while the pathogenic roles of its protein isoforms remain unclarified. We developed novel isoform‐specific antibodies and genetic knockdown (KD) of putative ASPM isoforms, whereby we uncovered that the levels of ASPM isoform 1 (iI) and ASPM‐iII are variably upregulated in PDAC cells. ASPM isoforms show remarkably different subcellular locations; specifically, ASPM‐iI is exclusively localized to the cortical cytoplasm of PDAC cells, while ASPM‐iII is predominantly expressed in cell nuclei. Mechanistically, ASPM‐iI co‐localizes with disheveled‐2 and active β‐catenin as well as the stemness marker aldehyde dehydrogenase‐1 (ALDH‐1), and its expression is indispensable for the Wnt activity, stemness, and the tumorigenicity of PDAC cells. By contrast, ASPM‐iII selectively regulates the expression level of cyclin E and cell cycle progression in PDAC cells. The expression of ASPM‐iI and ASPM‐iII displays considerable intratumoral heterogeneity in PDAC tissues and only that of ASPM‐iI was prognostically significant; it outperformed ALDH‐1 staining and clinico‐pathological variables in a multivariant analysis. Collectively, the distinct expression patterns and biological functions of ASPM isoforms may illuminate novel molecular mechanisms and prognosticators in PDAC and may pave the way for the development of therapies targeting this novel oncoprotein. © 2019 The Authors. *The Journal of Pathology* published by John Wiley & Sons Ltd on behalf of Pathological Society of Great Britain and Ireland.

## Introduction

Pancreatic ductal adenocarcinoma (PDAC) is a highly lethal and treatment‐resistant malignancy and the third leading cause of cancer‐related death in the USA 1. The majority of patients with PDAC present with locally advanced or metastatic disease and less than 20% of tumors are operable at diagnosis. Even with curative‐intent surgery, the majority of patients ultimately developed recurrent or metastatic diseases 2, 3. Despite recent advances in nanoparticle‐formulated chemotherapy, such as albumin‐bound paclitaxel and liposome‐encapsulated irinotecan, and immunotherapy, only a small fraction of patients with PDAC respond to these new types of therapies and the survival benefit gained from them is very limited. Further improvements in the prognosis of patients with PDAC will rely on elucidating the pathogenetic and molecular basis of tumor aggressiveness and progression, based on which pathway‐ and mechanism‐informed patient stratification systems and rational therapeutic strategies can be developed and deployed in the clinic.

A prevailing new paradigm of tumorigenesis proposes that only a subset of tumor cells with stem‐like properties, termed ‘tumor‐initiating cells (TICs)’ or ‘cancer stem cells (CSCs)’, has the ability to self‐renew and sustain tumorigenesis 4. CSCs have been found to exist in leukemia and multiple solid tumors 5, 6, 7, 8, 9. In PDAC, the cells that co‐express the surface markers CD44 and CD133 or CD44, CD24, and epithelial‐specific antigen have been shown to be enriched for pancreatic CSCs (panCSCs) 7, 10, 11. PanCSCs can also be enriched by selecting for their high aldehyde dehydrogenase 1 (ALDH‐1) activity, and the presence of ALDH‐1‐positive panCSCs has been associated with poor prognosis in patients with PDAC 12. Interestingly, ALDH‐1 seems to define a population of panCSCs that are more tumorigenic than those defined by CD133 or CD44 and CD24 13. Consistently, the presence of ALDH‐positive tumor cells in the circulation was associated with worse survival in patients with PDAC 14.

**Figure 1:**
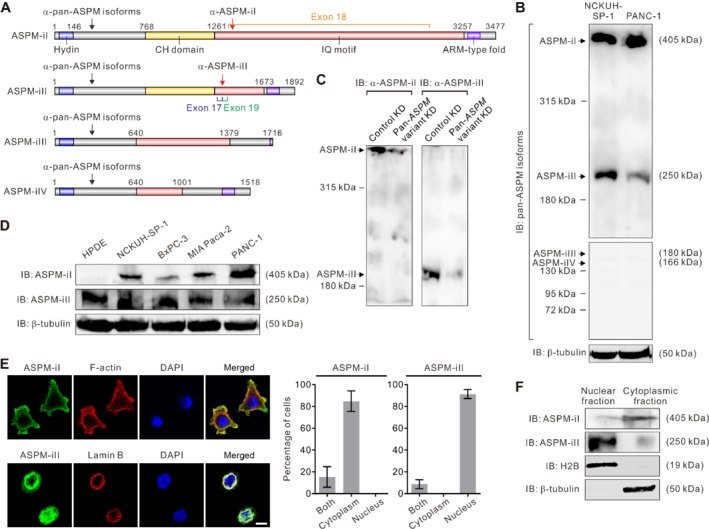
The expression pattern of ASPM isoforms in PDAC cells. (A) Schematic representation of different ASPM isoforms and their putative domains. Arrows indicate the locations of the immunogens of the pan‐isoform and the isoform‐specific anti‐ASPM antibodies. (B) Immunoblotting (IB) analysis using a pan‐ASPM‐isoform antibody showing the expression pattern of the putative ASPM isoforms in primary PDAC NCKUH‐SP‐1 cells and the PDAC line PANC‐1 cells. (C) IB analysis of ASPM‐iI (∼405 kDa) and ASPM‐iII (∼250 kDa) in NCKUH‐SP‐1 cells with stable knockdown (KD) of *ASPM* expression mediated by lentivirus‐mediated transduction of an shRNA targeting all putative *ASPM* variants. (D) IB analysis of ASPM‐iI and ASPM‐iII in normal‐like HPDE cells, primary NCKUH‐SP‐1 cells, and PDAC lines BxPC‐3, MIA Paca‐2, and PANC‐1 cells, using isoform‐specific antibodies. (E) Representative immunofluorescence (IF) images showing the differential subcellular localization of ASPM‐iI and ASPM‐iII in NCKUH‐SP‐1 cells. Scale bar = 10 μm. Right: quantification of the subcellular localization. (F) IB showing the protein abundance levels of ASPM‐iI and ASPM‐iII in the nuclear and the cytoplasmic fractions of NCKUH‐SP‐1 cells. Histone 2B (H2B) and β‐tubulin were included as the loading controls of nuclear and cytoplasmic fractions, respectively.

Whilst panCSCs have been implicated in the aggressiveness and the progression of human PDAC, the molecular regulators driving their existence and stemness properties remain unclarified. Recently, a novel co‐regulator of canonical Wnt–β‐catenin signaling, abnormal spindle‐like microcephaly‐associated (ASPM), was shown to regulate panCSCs and their stemness properties and tumorigenic potential 11. Several independently established prognostic gene signatures of PDAC also include ASPM as one of the marker genes 11, 15, 16. It has been predicted that several putative splicing variants of the *ASPM* transcripts, which encode the protein isoforms consisting of 3477 (isoform 1), 1892 (isoform 2), 1389 (isoform 3), and 1062 amino acids (isoform 4), respectively, may exist in normal and malignant human tissues 17. Compared with the largest isoform 1 (ASPM‐iI), the three smaller ASPM isoforms (isoforms 2–4) lack some of the functional domains of ASPM‐iI, such as the IQ (isoleucine and glutamine) motifs and the calponin‐homology (CH) domain (Figure 1A), raising the possibility that the different ASPM isoforms may play differential roles in normal and malignant cells. We sought, therefore, to determine the expression patterns of ASPM isoforms in human PDAC cells and tissues, and to assess whether they contribute differentially to the tumorigenicity of PDAC cells and the pathogenesis of PDAC. Our findings may illuminate novel molecular mechanisms and prognosticators in PDAC and may pave the way for the development of ASPM‐/Wnt‐/stemness‐targeted therapeutics.

## Materials and methods

### Cell culture

NCKUH‐SP‐1 cells were freshly isolated from the malignant ascites of a patient with metastatic PDAC. PANC‐1, BxPC‐3, and MiaPaCa‐2 cells (American Type Culture Collection, Manassas, VA, USA) were maintained in DMEM (Invitrogen, Carlsbad, CA, USA) supplemented with 10% fetal bovine serum and antibiotics. Human pancreatic ductal epithelial (HPDE) cells (a gift from M‐S Tsao, Ontario Cancer Institute, Toronto, ON, Canada) were propagated on tissue culture plastics as previously described 18, 19.

### Antibody production and validation

To probe the protein expression of all the putative ASPM isoforms (Figure 1A), we raised a rabbit polyclonal antibody using a synthetic peptide shared by all the isoforms (amino acids 394–493), which we designated as the ‘pan‐isoform’ anti‐ASPM antibody. To specifically detect ASPM‐iI (NCBI RefSeq: NP_060606.3) and isoform 2 (ASPM‐iII; NCBI RefSeq: NP_001193775.1), we raised rabbit polyclonal antibodies using synthetic peptide epitopes specific for ASPM‐iI and ASPM‐iII, respectively. Details of the immunogen design and the antibody validation are described in supplementary material, Supplementary materials and methods.

### Immunoblotting (IB) and co‐immunoprecipitation (co‐IP)

IB analysis and co‐IP experiments were performed according to standard protocols. Antibodies used for IB experiments included anti‐dishevelled (Dvl)‐2 (H‐75; Santa Cruz, Dallas, TX, USA), anti‐cyclin E (GeneTex, Hsinchu City, Taiwan), and anti‐non‐phospho (active) β‐catenin (D13A1) and anti‐caspase‐3 (both from Cell Signaling, Danvers, MA, USA). A goat anti‐rabbit IgG (Jackson ImmunoResearch, West Grove, PA, USA) was used in conjunction with the polyclonal antibodies raised for the immunodetection of ASPM isoforms as described above. Proteins were revealed following SDS/PAGE and immunoblotting with the indicated antibodies.

### Gene expression manipulations

The specific knockdown (KD) of the expression of *ASPM* transcript variant 1 (*ASPM*‐vI; NCBI RefSeq: NM_018136.4; Figure 1A), which encodes *ASPM*‐iI, was achieved by an *ASPM* exon 18 (unique to *ASPM*‐vI)‐specific small hairpin RNA (shRNA). The specific KD of the expression of *ASPM*‐vII (NCBI RefSeq: NM_001206846) was carried out using three different RNAi target sequences that cross the splicing junction site connecting exons 17 and 19 (unique to *ASPM*‐vII). The construction of the lentiviral vector expressing the selected shRNA and the virus production procedure are described in supplementary material, Supplementary materials and methods. Nonspecific KD of all putative *ASPM* variants, including *ASPM*‐vI and *ASPM*‐vII, was achieved by lentivirus‐mediated RNAi using a commercial shRNA oligonucleotide (clone TRCN0000118905) in the lentivector pLKO.1‐puro (MISSION shRNA lentiviruses; Sigma‐Aldrich, St Louis, MO, USA).

### Luciferase reporter assay

For luciferase reporter analysis, cells were transduced with Cignal Lenti TCF/LEF Reporter (Qiagen, Venlo, The Netherlands). Following stimulation of the cells with recombinant human Wnt‐3a (250 ng/ml for 16 h; R&D Systems, Minneapolis, MN, USA) or vehicle, the reporter activity was measured by using the ONE‐Glo™ Luciferase Assay System (Promega, Madison, WI, USA).

### Flow cytometry and tumorsphere assays

The ALDEFLUOR assay (StemCell Technologies, Vancouver, Canada) was performed according to the manufacturer's recommendations. Flow cytometry used a FACSCanto™ II flow cytometer (BD Biosciences). The tumorsphere assay was performed as described previously 20. The limiting dilution assay (LDA) is described in supplementary material, Supplementary materials and methods.

### Immunofluorescence (IF) and immunohistochemistry (IHC) analyses

IF staining of cells grown on culture plastics was performed using standard protocols as described in supplementary material, Supplementary materials and methods. Specific antibodies against ASPM‐iI or ASPM‐iII (described above), Dvl‐2 (H‐75; Santa Cruz), active (non‐phospho) β‐catenin (D13A1; Cell Signaling Technology), and ALDH‐1 (44/ALDH; BD Transduction Laboratories, San José, CA, USA) were used for the staining. For IHC analysis, we acquired formalin‐fixed, paraffin‐embedded tissues of PDAC from 50 patients who had received tumor resection at the National Cheng Kung University Hospital (NCKUH) during 2015–2018 in conformity with Institutional Review Board‐approved protocols. Informed consent was obtained from all the patients. The clinical characteristics of the patient cohort are presented in supplementary material, Table S1. IHC analysis was conducted as described in supplementary material, Supplementary materials and methods. Confocal and IF imaging was performed using a Nikon Digital Eclipse C1 confocal microscope system and an Olympus IX81 fluorescence microscope, respectively. IHC and IF staining were independently evaluated and verified by two expert pathologists (WYC and CTL) in a randomized manner.

### Statistical analysis

The statistical programming language R (https://http://cran.r-project.org) and SPSS 10.0 software (SPSS, Chicago, IL, USA) were used to conduct the statistical analysis of our data. Survival curves were generated using the Kaplan–Meier method. The curves were plotted and compared using the log‐rank test in Prism 6.01 software (GraphPad Software, San Diego, CA, USA). A cut‐off value that best discriminated between groups with respect to outcome was determined using the concordance index, where an index of 1.0 is perfect discrimination 21.

## Results

### Differential expression patterns of ASPM isoforms in PDAC cells and tissues

IB analysis performed using a pan‐ASPM‐isoform antibody (Figure 1A, black arrows) on extracts of primary NCKUH‐SP‐1 cells (which were freshly isolated from a patient with metastatic PDAC) and the established PDAC line PANC‐1 cells revealed that ASPM‐iI (∼405 kDa) and ASPM‐iII (∼250 kDa), which lacks the 67 IQ domains carried by exon 18 of the *ASPM* gene, were the two major ASPM isoforms detectable in these cells (Figure 1B). The other isoforms, including ASPM iIII and ASPM iIV, were expressed at negligible levels in PDAC cells. In order to specifically detect ASPM‐iI and ASPM‐iII, we raised rabbit polyclonal antibodies using peptide immunogens specific to ASPM‐iI and ASPM‐iII, respectively (Figure 1A, red arrows). Specifically, the 16‐amino acid (aa) immunogen used to raise the ASPM‐iI‐specific antibody was selected from an aa sequence encoded by exon 18 of the *ASPM* gene, which exists exclusively on ASPM‐iI (Figure 1A). Since the sequence of ASPM‐iII differs with that of ASPM‐iI only in the region encoded by exon 18 of the *ASPM* gene 17, we designed and synthesized a short 9‐aa peptide (SLIQAMWRR), spanning the region encoded by the splicing junction of exons 17 and 19 of the *ASPM* gene, as an immunogen to ensure that the antibody raised was specific to this small epitope existing only on ASPM‐iII. We confirmed that each of these antibodies detected proteins of the expected size and reaffirmed their specificity by knocking down the expression of *ASPM* based on lentivirus‐mediated transduction of an shRNA targeting all the putative transcript variants (pan‐*ASPM*‐variant KD), which led to much reduced abundance levels of both ASPM‐iI and ASPM‐iII being detected using their respective antibodies (Figure 1C). These novel isoform‐specific antibodies provided an unprecedented opportunity for evaluating the expression patterns of different ASPM isoforms in PDAC cells and tissues. Intriguingly, ASPM‐iI was found to be overexpressed in primary NCKUH‐SP‐1 cells and a panel of PDAC lines at variable levels much higher than that of normal‐like HPDE cells (Figure 1D). By contrast, the protein abundance level of ASPM‐iII was also variable, while it did not increase significantly in PDAC cells. The expressional variability of ASPM‐iI and ASPM‐iII did not seem to correlate with each other among PDAC cells.

We undertook profiling of the spatial distribution of *ASPM*‐vI and *ASPM*‐vII in PDAC cells using IF analysis. Unexpectedly and surprisingly, ASPM‐iI was found to be specifically and exclusively expressed in the cytoplasm, especially the cortical region, wherein it co‐localizes with cortical actin, in PDAC cells (Figure 1E, upper panels). By contrast, ASPM‐iII was both nuclear and cytoplasmic but was predominantly present in cell nuclei (Figure 1E, lower panels). The differential subcellular localization of ASPM‐iI and ASPM‐iII was confirmed by the IB analyses on the cell nucleus‐ and the cytoplasm‐enriched protein lysates of the cells (Figure 1F). Of note, whilst a fraction of ASPM‐iII was cytoplasm‐localized, it did not interact with ASPM‐iI in a co‐IP analysis (supplementary material, Figure S2). Taken together, our findings identified ASPM‐iI and ASPM‐iII as the abundantly expressed ASPM isoforms in PDAC cells, which localized to different subcellular compartments and did not associate with each other.

**Figure 2:**
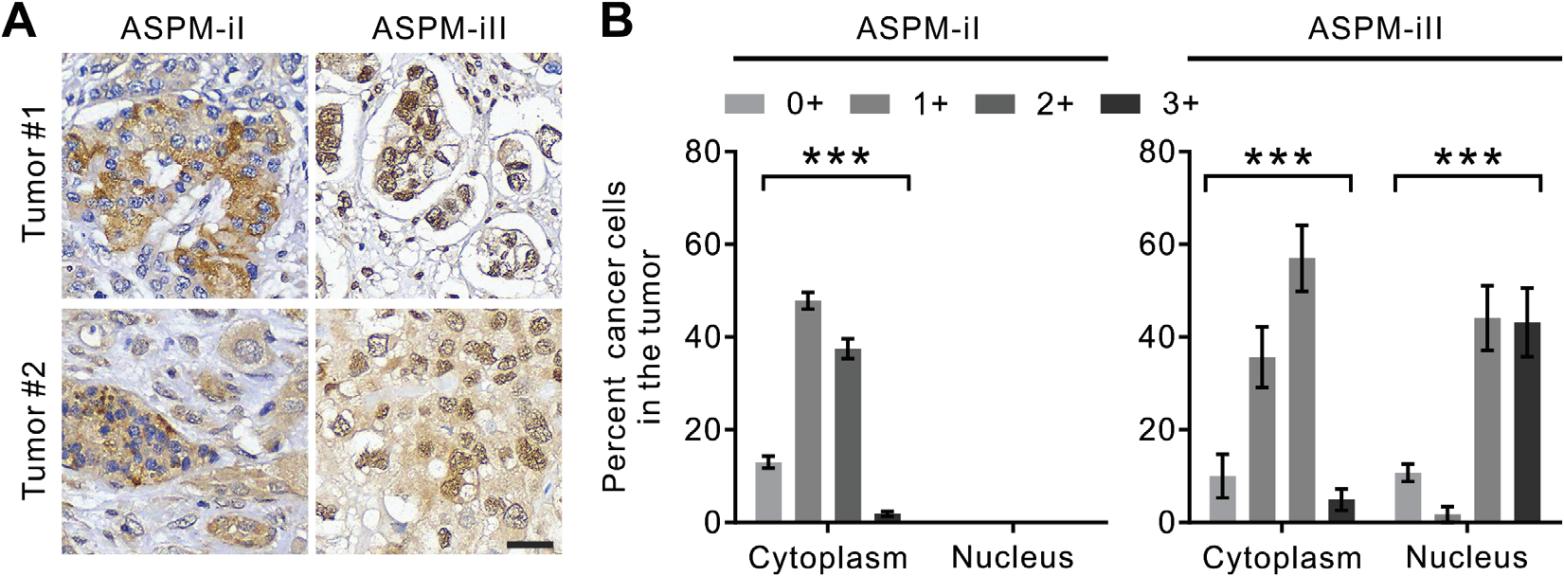
The differential expression patterns of ASPM‐iI and ASPM‐iII in PDAC tissues. (A) IHC staining of ASPM‐iI and ASPM‐iII in representative human PDAC tissues (tumor #1 and tumor #2; 200× magnification). Scale bar = 30 μm. (B) Bar charts showing the distribution of the single‐cell staining intensities (0+ to 3+) of ASPM‐iI and ASPM‐iII in human PDAC tissues. Mean ± SEM (*n* = 50). ***p < 0.001.

To verify the differential expression patterns of ASPM‐iI and ASPM‐iII in human PDAC tissues, we performed IHC staining of the whole‐tumor tissue sections from 50 patients with resected PDAC (supplementary material, Table S1). Consistently, we showed that ASPM‐iI was present specifically in the cytoplasm of the tumor cells, while ASPM‐iII was either mainly nuclear‐localized (tumor #1) or present in both nuclear and cytoplasmic compartments (tumor #2; Figure 2A). Importantly, both isoforms showed a considerable (*p* < 0.001) expressional heterogeneity within the same tumor (Figure 2B).

### ASPM isoforms differentially interact with Dvl‐2 and cyclin E in PDAC cells

**Figure 3:**
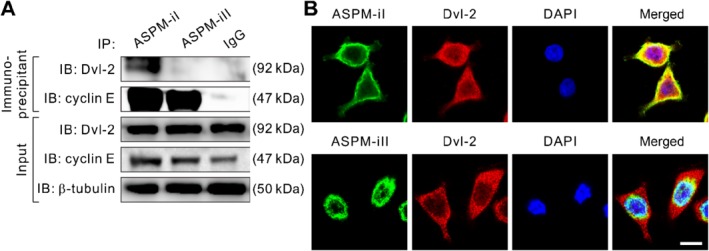
ASPM‐iI and ASPM‐iII differentially interact with Dvl‐2 and cyclin E. (A) Co‐IP of ASPM‐iI or ASPM‐iII with Dvl‐2 or cyclin E using ASPM‐isoform‐specific antibodies or control IgG in NCKUH‐SP‐1 cells. β‐Tubulin was included as a loading control. (B) Representative confocal images showing the strong co‐localization (yellow) of ASPM‐iI (green) with Dvl‐2 (red) in NCKUH‐SP‐1 cells. Nuclei were counterstained with DAPI (blue). Note that ASPM‐iII does not co‐localize with Dvl‐2 (bottom). Scale bar = 10 μm.

We have previously shown that ASPM augments canonical Wnt signaling by positively regulating critical upstream Wnt mediators, including Dvl proteins and β‐catenin, in PDAC and prostate cancer cells 11, 22. The dramatically different subcellular localizations of ASPM‐iI and ASPM‐iII implied that they may have distinct roles in the molecular regulation of PDAC cells. Indeed, co‐IP experiments in PDAC cell lysates using isoform‐specific antibodies revealed that ASPM‐iI strongly associated with Dvl‐2, an upstream regulator in Wnt signaling 23, in PDAC cells (Figure 3A). IF analysis confirmed the strong co‐localization of ASPM‐iI with Dvl‐2 (Figure 3B, top). By contrast, ASPM‐iII did not associate or co‐localize with Dvl‐2 (Figure 3B, bottom).

ASPM is a large and pleiotropic protein and it has been shown recently that ASPM is involved in regulation of the cell cycle, especially the late G1 cell‐cycle regulator cyclin E/Cdk2 complex, by stabilizing cyclin E protein in neuron progenitor cells 24. Congruently, we found that both ASPM‐iI and ASPM‐iII interacted with cyclin E in PDAC cells (Figure 3A).

### ASPM‐iI and ASPM‐iII differentially regulate Wnt signaling, cancer stemness, and cell cycle progression in PDAC cells

**Figure 4:**
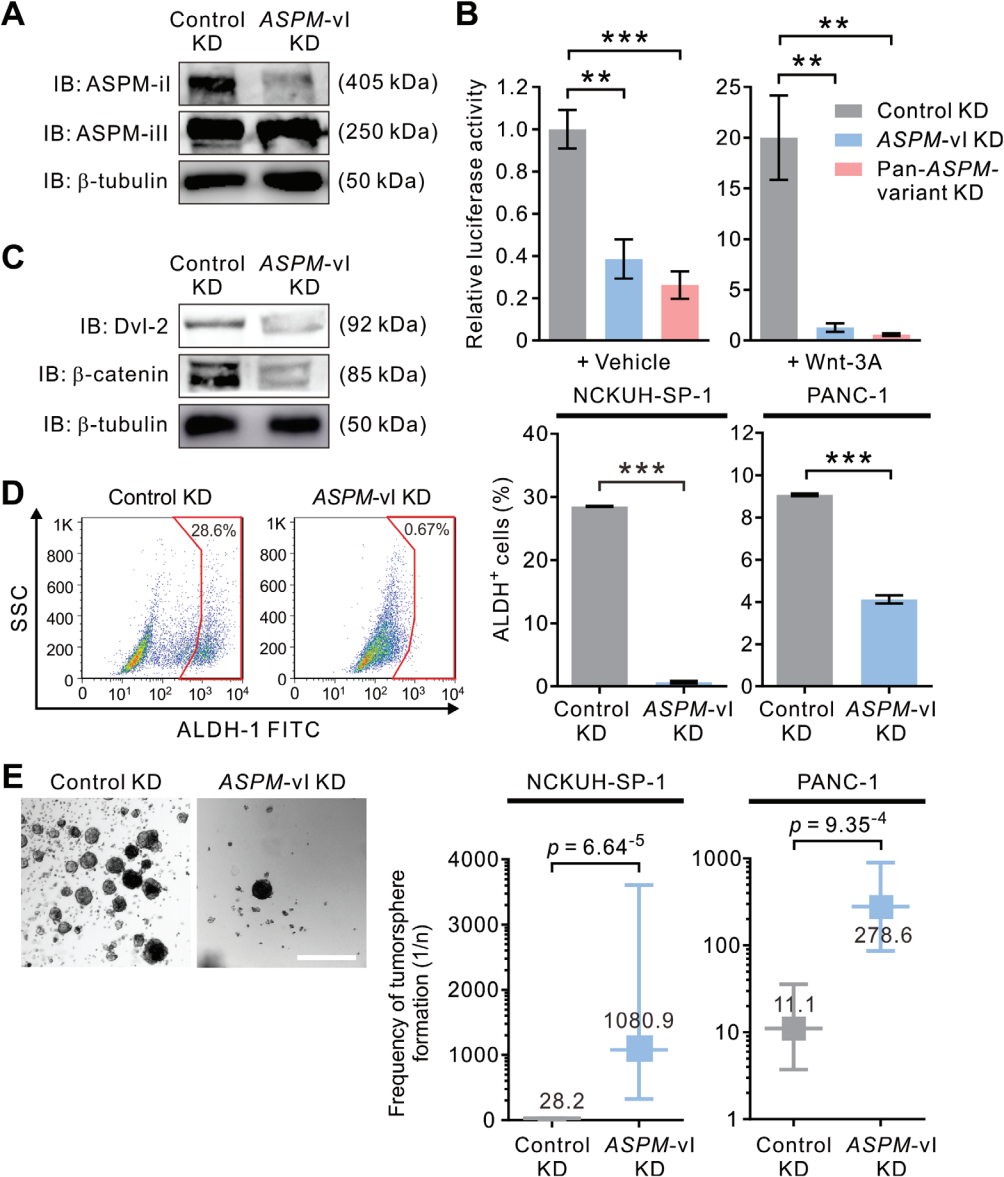
ASPM‐iI specifically regulates Wnt activity and the stemness properties of PDAC cells. (A) IB showing the effect of the isoform‐specific KD of *ASPM*‐vI on the protein abundance levels of ASPM‐iI and ASPM‐iII in NCKUH‐SP‐1 cells. (B) Relative Wnt‐specific luciferase expression in control KD or *ASPM*‐vI KD and Wnt‐3a (250 ng/ml × 16 h)‐treated NCKUH‐SP‐1 cells. A non‐target shRNA (control shRNA) and an shRNA targeting all *ASPM* variants (clone TRCN0000118905; pan‐*ASPM*‐variant KD) were used as controls. (C) IB showing that specific KD of the expression of *ASPM*‐vI reduced the protein abundance levels of Dvl‐2 and β‐catenin in NCKUH‐SP‐1 cells. (D) *ASPM*‐vI KD diminished the population of ALDH^+^ cells in NCKUH‐SP‐1 cells. Representative flow cytometry plots showing the pattern of ALDH activity in control KD or *ASPM*‐vI KD NCKUH‐SP‐1 cells, with the frequency of the boxed ALDH^+^ cell population as a percentage of cancer cells shown. Bottom: the percentage of ALDH^+^ cells. (E) Representative phase contrast images of control KD or *ASPM*‐vI KD NCKUH‐SP‐1 cells. Scale bar = 200 μm. Right: limiting dilution assay demonstrating the tumorsphere‐forming efficacy of control KD or *ASPM*‐vI KD NCKUH‐SP‐1 cells. Mean ± SEM (*n* = 6 in each group). **p < 0.01; ***p < 0.001.

To gain mechanistic insights into the different functional roles of ASPM isoforms in PDAC cells, we sought to specifically downregulate the transcript levels of *ASPM*‐vI (encoding ASPM‐iI) and *ASPM*‐vII (encoding ASPM‐iII) in PDAC cells using lentivirus‐mediated and isoform‐specific RNA interference. We were able to specifically knock down the expression of *ASPM*‐vI to a level of approximately 25% of that of the cells infected with the control shRNA virus (Figure 4A). However, we failed to specifically knock down the expression of *ASPM*‐vII without affecting that of *ASPM*‐vI in spite of the use of three RNAi target sequences that cross the splicing junction site connecting exons 17 and 19 (unique to *ASPM*‐vII). Notwithstanding this technical difficulty, we were able to demonstrate that the specific KD of *ASPM*‐vI expression (*ASPM*‐vI KD) dramatically attenuated Wnt pathway activation in PDAC cells to an extent comparable to that induced by a pan‐ASPM‐variant KD (Figure 4B). Consistent with this, *ASPM*‐vI KD significantly reduced the protein abundance level of Dvl‐2 as well as its downstream effector β‐catenin (Figure 4C), reaffirming the previously reported role of ASPM in the Wnt–Dvl–β‐catenin signaling axis 11, 25. Previously, we reported the critical role of ASPM in the regulation of CSCs in PDAC and prostate cancer 11, 25. Consistent with this, *ASPM*‐vI KD led to a substantial reduction of ALDH‐1^+^ panCSCs (Figure 4D) and inhibited the tumorsphere‐forming ability (an *in vitro* surrogate of cancer stemness) of PDAC cells (Figure 4E).

**Figure 5:**
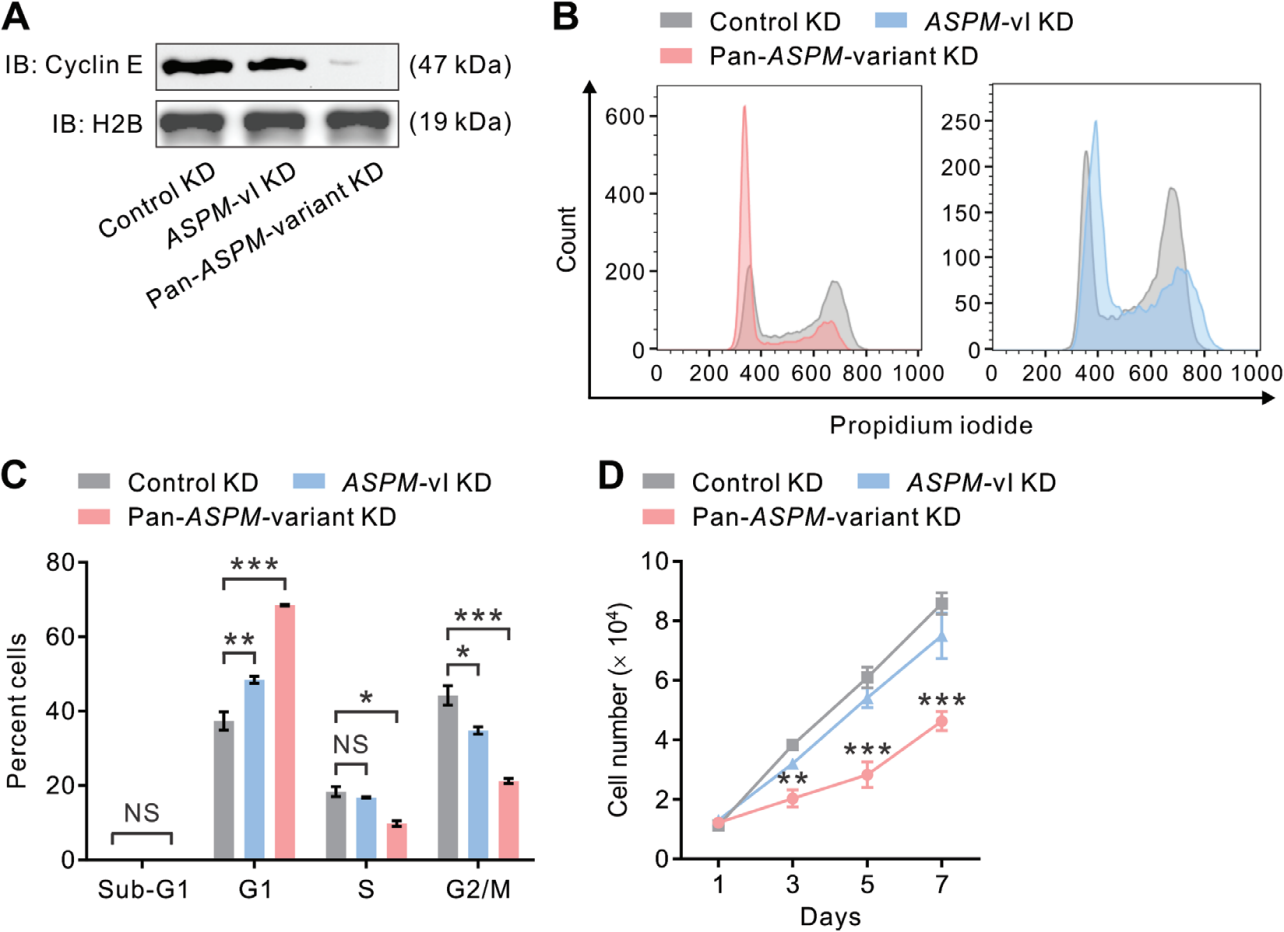
ASPM‐iII selectively regulates cyclin E and cell cycle progression in PDAC cells. (A) IB showing the protein abundance level of cyclin E in NCKUH‐SP‐1 cells with control KD, *ASPM*‐vI KD or pan‐*ASPM*‐variant KD mediated by lentivirus‐mediated RNA interference. H2B was included as a loading control. (B) Cell cycle distributions of NCKUH‐SP‐1 cells with control KD or pan‐*ASPM*‐variant KD. (C) The percentage of cells in the different phases in the cell cycle or those with a sub‐G1 DNA content in NCKUH‐SP‐1 cells with control KD, *ASPM*‐vI KD or pan‐*ASPM*‐variant KD. NS, not significant. Mean ± SEM (*n* = 3 in each group). *p < 0.05; **p < 0.01; ***p < 0.001. (D) Line graphs showing the proliferative rate of NCKUH‐SP‐1 cells with control KD, *ASPM*‐vI KD or pan‐*ASPM*‐variant KD. Mean ± SEM (*n* = 3 in each group). **p < 0.01; ***p < 0.001 versus control KD.

Although our preceding co‐IP analysis revealed that both ASPM‐iI and ASPM‐iII interacted with cyclin E (Figure 3A), the observations that ASPM‐iI is exclusively cytoplasm‐localized while ASPM‐iII is mainly nuclear‐localized in PDAC cells (Figure 1E) raised the possibility that ASPM‐iII plays a significant role in the regulation of cyclin E and the cell cycle in PDAC cells. ASPM has been shown to interact directly with cyclin E and thereby inhibit its protein ubiquitination and increase its stability in a Cdk2‐independent manner 24. Indeed, whilst the pan‐*ASPM*‐variant KD targeting the transcripts of both *ASPM*‐vI and *ASPM*‐vII dramatically reduced the protein abundance level of cyclin E, *ASPM*‐vI KD did not significantly affect the expression level of cyclin E (Figure 5A). In keeping with the biochemical data, pan‐*ASPM*‐variant KD induced a significant increase in the cells in G1 phase (averaged 37.4% in control KD to 68.5% in pan‐*ASPM‐*variant KD; *p* < 0.001) and a decrease in the cells in S phase (averaged 18.4% to 9.8%; *p* < 0.01), indicating a partial cell‐cycle arrest at the G1 phase, in PDAC cells (Figure 5B,C). By contrast, *ASPM*‐vI KD only slightly increased the percentage of cells in the G1 phase. Concordantly, pan‐*ASPM*‐variant KD markedly reduced the proliferation of PDAC cells, whereas *ASPM*‐vI KD had an insignificant effect on cell proliferation (Figure 5D). Moreover, neither pan‐*ASPM*‐variant nor *ASPM*‐vI KD led to the appearance of cells with a sub‐G1 DNA content or those expressing cleaved caspase‐3 (Figure 5C and supplementary material, Figure S3). Taken together, these data suggest that ASPM‐iI and ASPM‐iII are functionally distinct and regulate different molecular processes and biology in PDAC cells.

### ASPM‐iI as a surrogate marker of Wnt activity and stemness and a prognosticator in PDAC

**Figure 6:**
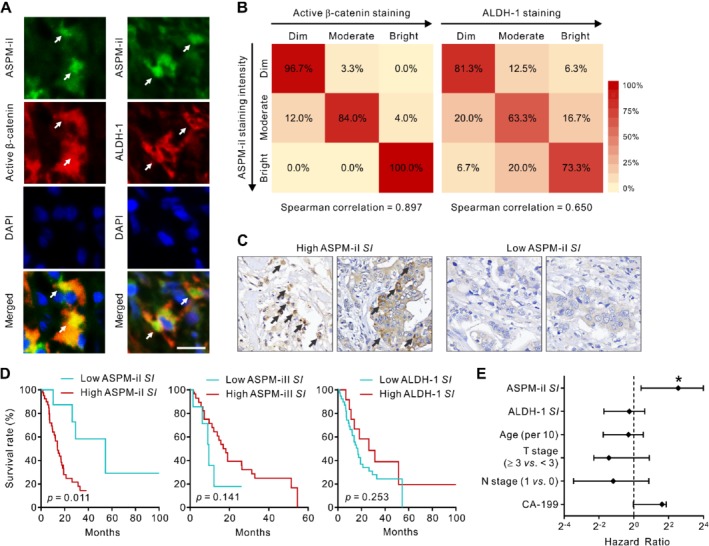
ASPM‐iI co‐localizes with active β‐catenin and ALDH‐1 and is prognostically significant in PDAC. (A) Representative IF images showing the co‐localization (yellow; arrows) of ASPM‐iI (green) with active β‐catenin (red, left panels) or ALDH‐1 (red, right panels) in human PDAC tissues. Nuclei were counterstained with DAPI (blue). Scale bar = 25 μm. (B) Heatmaps illustrating the correlation of the staining intensity of ASPM‐iI with that of active β‐catenin or ALDH‐1 in PDAC tissues (*n* = 50). (C) Representative IHC images (400× original magnification) showing the tumors with high or low ASPM‐iI SI. Arrows indicate tumor cells with a high (≥ 2+) staining intensity of ASPM‐iI. (D) The staining pattern of ASPM‐iI is prognostically significant in PDAC. Kaplan–Meier survival curve comparing overall survival of the patients with resected PDAC (*n* = 50) stratified according to the staining index (SI; the percentage of tumor cells expressing a moderate‐to‐high staining intensity) of ASPM‐iI, ASPM‐iII, or ALDH‐1 within the tumors. The cut‐off value of each SI was determined using the concordance index. (E) Forest plots showing hazard ratios (with 95% confidence limits) of death according to the SI of ASPM‐iI or ALDH‐1 or clinico‐pathological criteria in a Cox proportional‐hazards analysis. *p < 0.05.

Previously, the staining intensity of ASPM as determined by IHC analysis using a pan‐ASPM‐isoform antibody has been shown to correlate with poor prognosis in patients with PDAC 11. To gain clinical relevance of the above findings and to compare the prognostic value of different ASPM isoforms, we examined the tumor tissues for the staining patterns of ASPM‐iI, ASPM‐iII, active β‐catenin (a marker of activated Wnt–β‐catenin signaling), and the stemness marker ALDH‐1, which has been shown to be prognostically important in PDAC 12, at the single‐cell resolution in a cohort of 50 consecutive PDAC patients who received surgical resection of their primary tumors (supplementary material, Table S1). IF analysis on these tumors verified the significant co‐localization of ASPM‐iI and active β‐catenin or ALDH‐1 (Figure 6A). Indeed, the staining intensity of ASPM‐iI among single cancer cells within the same tumor correlates significantly with that of active β‐catenin (Spearman correlation = 0.897; Figure 6B). The staining intensity of ASPM‐iI also correlates significantly with that of ALDH‐1, albeit to a lesser extent (Spearman correlation = 0.650).

Given the considerable intratumoral heterogeneity in the expression of ASPM‐iI and ASPM‐iII in PDAC (Figure 2B), and that only a small subset of CSCs may serve as the driving force of PDAC progression and aggressiveness 7, 10, 11, 12, we investigated the prognostic value of the percentage of cells expressing high levels of ASPM‐iI or ASPM‐iII in PDAC. We meticulously scored the staining intensities of ASPM‐iI, ASPM‐iII, and ALDH‐1 at the single‐cell resolution in multiple tissue sections from the same tumors (three tissue sections per tumor; at least 100 tumor cells counted per section) and stratified the patients according to the percentage of tumor cells in their tumors exhibiting a high (≥ 2+) staining intensity of each of these markers, which was denoted as the ‘staining index (SI)’ of said marker. We classified the patients according to the cut‐off value determined by the statistically stringent concordance index. Patients with tumors with a high ASPM‐iI SI (≥ 1.5%; Figure 6C) had a significantly higher risk of death following surgery than those with tumors with a low ASPI‐iI SI (log‐rank *p* = 0.011; Figure 6D, top; Cox regression *p* = 0.018). By contrast, the SI of ASPM‐iII or ALDH‐1 was not prognostically significant (Cox regression *p* = 0.697 for ASPM‐iII; Cox regression *p* = 0.201 for ALDH‐1; Figure 6D). A multivariable analysis of survival using Cox proportional hazard modeling confirmed that ASPM‐iI SI is a significant and independent predictor of survival (*p* = 0.015); specifically, patients with tumors with a high ASPM‐iI SI had an approximately six‐fold increase in the long‐term risk of mortality than those with tumors with a low ASPM‐iI SI (hazard ratio = 5.863; 95% confidence interval = 1.338–15.896; Figure 6E and supplementary material, Table S2). Importantly, ASPM‐iI SI significantly outperforms standard clinico‐pathological criteria and ALDH‐1 SI in the prognostic prediction.

## Discussion

PanCSCs have been considered as the driver of PDAC aggressiveness and disease progression 7, 10, 11, 12, 13. Therapeutics targeting panCSCs with a variety of mechanisms of action have been developed, including the combined inhibition of sonic hedgehog and mTOR signaling 26, the small molecule inhibitor of signal transducer and activator of transcription (STAT)‐3, napabucasin 27, and the Notch signaling inhibitors tarextumab and demcizumab 28, 29. Despite the encouraging pre‐clinical efficacy of these novel anti‐CSC agents, they failed a series of phase 2 and phase 3 clinical trials in PDAC and other types of solid cancers due to toxicity and/or efficacy issues, calling into question whether or not their targets are *de facto* CSC‐specific. These clinical setbacks highlight the need of identifying more specific regulators and/or markers of cancer stemness and panCSCs, which can serve as better therapeutic targets in PDAC or biomarkers and companion diagnostics that can guide the patient selection and the design of future clinical trials 30.

There is now substantial evidence that Wnt signaling plays an important role in the tumorigenesis and the aggressiveness of PDAC 31, 32. For instance, PDAC cells in ascites and the blood circulation express high levels of Wnt‐2 31. A high Wnt/β‐catenin transcriptional activity has been linked to lympho‐vascular invasion in human PDAC 20. PDAC cells upregulate the expression of Wnt‐7B, Wnt‐2, and ataxia–telangiectasia group D complementing (ATDC), which stabilizes the Dvl‐2 protein to promote Wnt signaling 20. Interestingly and importantly, as opposed to the bulk PDAC cells, panCSCs seem to exploit a different set of molecular mechanisms to augment Wnt signaling to maintain their population and stemness properties. For instance, the novel oncoprotein family with sequence similarity 83 member A (FAM‐83A) promotes PDAC stemness and chemoresistance by activating Wnt signaling 33. PanCSCs also upregulate the expression of the novel oncoprotein ASPM, which augments Wnt signaling by positively regulating Dvl‐2 and β‐catenin 11, 22.

Among the reported markers of panCSCs, only ALDH‐1 has been unambiguously linked to the prognosis of patients with PDAC, based on a clinical correlative study in a large patent cohort (*n* = 269) 12. The prognostic roles of the surface markers CD44, CD24, and/or CD133 are less well defined 34, 35. However, these surrogate markers of cancer stemness are neither CSC‐exclusive nor pathway‐ or biology‐informed, precluding their utility as biomarkers or companion diagnostics for the development of rational therapeutics. In this regard, the panCSC regulator ASPM, which we showed has a strong and robust prognostic role in PDAC, may provide the first pathway (Wnt)‐ and biology (cancer stemness)‐informed prognosticator in PDAC. In the present study, we went one step further to elucidate the roles of different ASPM isoforms in Wnt signaling and PDAC stemness, and also performed meticulous clinical correlative analyses to verify their respective prognostic significance. Surprisingly, of the major ASPM isoforms expressed in PDAC, only ASPM‐iI interacts with Dvl‐2 and activates canonical Wnt signaling and maintains the population of panCSCs. The distinct role of ASPM‐iI in Wnt signaling and cancer stemness can be attributed to its predominant subcellular localization in the cytosol, where most of the upstream Wnt regulators are localized, as well as the existence of the large protein fragment (1975 amino acids) encoded by exon 18 of the *ASPM* gene (Figure 1A), which may presumably comprise the region interacting with Dvl‐2. By contrast, ASPM‐iII is mainly localized in cell nuclei; therefore, it predominantly interacts with cyclin E to regulate its stability, thereby likely mainly regulating the cell cycle progression, especially the G1/S transition, in PDAC cells (Figure 5B,C) 24. The different subcellular localization and molecular functions of ASPM‐iI and ASPM‐iII, together with the specific upregulation of the expression of ASPM‐iI but not that of ASPM‐iII in PDAC cells (Figure 1D), raised the possibility that ASPM‐iI may specifically contribute to PDAC progression and stemness, while ASPM‐iII may be predominantly responsible for the housekeeping function of ASPM, such as cell cycle and mitosis regulation. Further in‐depth molecular and mechanistic studies are still demanded to dissect the pleiotropic functions of this large and interesting protein.

In keeping with the differential biological functions of ASPM isoforms in PDAC cells, we demonstrated that ASPM‐iI, but not ASPM‐iII, co‐localizes with active β‐catenin and the panCSC marker ALDH‐1 in human PDAC tissues. Notably and importantly, the expression pattern of ASPM‐iI, as reflected by the ASPM‐iI SI, significantly outperformed that of ALDH‐1 and standard clinico‐pathologic variables in the prognostic prediction of PDAC patients. This finding supports the idea that a knowledge‐based approach may offer an opportunity to identify best‐performing biomarkers or classifiers that additionally provide pathogenetic information in cancer patients. Moreover, a pathway‐ and biology‐informed biomarker like ASPM‐iI SI may fulfill the need of a diagnostic assay that can help clinicians or investigators prognostically classify patients, whereby clinical trials related to the specific cancer biology can be conducted in a patient‐tailored manner. It should be noted, however, that the prognostic role of ASPM‐iI was determined in a relatively small (*n* = 50) cohort of PDAC patients, and requires further verification in an independent and larger patient cohort than the present one. Similarly, whether or not the SI of ASPM‐iII remains prognostically insignificant also awaits further investigation.

In conclusion, the current study identified the differential expression pattern, subcellular localization, as well as the molecular roles of different ASPM isoforms in PDAC cells. ASPM‐iI is specifically cytoplasmic‐localized and regulates Wnt signaling and panCSCs through its antagonism with the E3 ubiquitin ligase of Dvl‐2, whereas ASPM‐iII is mainly nuclear‐localized and regulates essential housekeeping functions of PDAC cells. The superior performance of ASPM‐iI staining in the prognostic prediction supports it as a novel pathway‐informed and Wnt‐related marker of cancer stemness as well as a clinically useful and immediately applicable prognosticator that can not only predict the outcome and survival of patients with resected PDAC but also guide future Wnt‐ and/or CSC‐targeted rational therapies.

## Author contributions statement

C‐CH and W‐YL carried out experiments and data analysis. T‐SC performed data collection and analysis. W‐YC and C‐TL were responsible for histopathological examination and scoring. Y‐SS provided patient data for the study, and collected and analyzed data. P‐JH and C‐RL analyzed data. Y‐CH provided patient data for the study and collected data. KKT conceived, designed, and supervised the study; analyzed data; wrote the original draft; and reviewed and edited the paper. All the authors approved the final version of the article as submitted and agreed to be accountable for all aspects of the work.

## Supporting information


**Supplementary materials and methods**

**Figure S1.** The performance of isoform‐specific anti‐ASPM antibodies in PDAC cells
**Figure S2.** ASPM‐iI and ASPM‐iII do not interact with each other in PDAC cells
**Figure S3.** Knock‐down (KD) of *ASPM* transcripts or *ASPM*‐vI expression does not induce apoptosis in PDAC cells

**Table S1.** Clinical characteristics of the patients in the NCKUH cohort
**Table S2.** Multivariate Cox regression model predicting overall survival by the ASPM‐iI *Staining Index* and clinico‐pathological criteria.
