# Supplementary material for: The differential distributions of ASPM isoforms and their roles in Wnt signaling, cell cycle progression, and pancreatic cancer prognosis
Source: J Pathol. 2019 Oct 23;249(4):498–508. doi: 10.1002/path.5341 (PMC6899738; doi:10.1002/path.5341)
Supplement: Supplementary file 1 — Supplementary materials and methods [file PATH-249-498-s003.docx]

**The differential distributions of ASPM isoforms and their roles in Wnt signaling, cell cycle progression, and pancreatic cancer prognosis**

Hsu C-C *et al. J Pathol* DOI: 10.1002/path.5341

**Supplementary materials and methods**

Reference numbers refer to the main text reference list

**Cell culture**

NCKUH-SP-1 cells were freshly isolated from the malignant ascites of a patient with metastatic PDAC at National Cheng Kung University Hospital (NCKUH, Tainan, Taiwan). The tumor tissue was acquired and used in conformity with Institutional Review Board-approved protocols. PANC-1, BxPC-3, and MiaPaCa-2 cells (American Type Culture Collection, Manassas, VA, USA) were maintained in DMEM (Invitrogen, Carlsbad, CA, USA) supplemented with 10% fetal bovine serum and antibiotics. Human pancreatic ductal epithelial (HPDE) cells (a gift from M-S Tsao, Ontario Cancer Institute, Toronto, ON, Canada), which are human papillomavirus-E6 and -E7 gene-immortalized and normal-like pancreatic ductal epithelial cells [18,19], were propagated on tissue culture plastics in Keratinocyte-SFM (Sigma-Aldrich, St Louis, MO, USA) supplemented with bovine pituitary extract, 10 ng/ml EGF, 0.5% horse serum, and antibiotics (Invitrogen). Cell line authentication was performed by the Bioresource Collection and Research Center (Hsinchu, Taiwan).

**Antibody production and validation**

To probe the protein expression of the four putative ASPM isoforms (Figure 1A), we raised a rabbit polyclonal antibody using a synthesized immunogen shared by all the isoforms (amino acids 394–493: NQFLKDNMAYMCTSQQTCKVPLSNENSQVPQSPEDWRKSEVSPRIPECQGSKSPKAIFEELVEMKSNYYSFIKQNNPKFSAVQDISSHSHNKQPKRRPIL), which we designated as the ‘pan-ASPM-isoform antibody’. To specifically detect ASPM isoform 1 (ASPM-iI; NCBI RefSeq: NP_060606.3) and isoform 2 (ASPM-iII; NCBI RefSeq: NP_001193775.1), we raised rabbit polyclonal antibodies using peptide immunogens specific for ASPM-iI (RRYSTRQRFLKLKYYS; encoded specifically by a sequence spaced within exon 18 of the *ASPM* gene) and ASPM-iII (SLIQAMWRR; spanning the region encoded by the splicing junction of exons 17 and 19 of the *ASPM* gene), respectively. We confirmed that the sequences used are unique to ASPM-iI and ASPM-iII, respectively, and do not align with any other known human genes by the BLAST sequence analysis. The specificity of the antibodies for ASPM-iI or ASPM-iII were verified by the subsequent immunoblotting (IB), co-immunoprecipitation (IP), and immunofluorescence (IF) analyses, with the use of the respective pre-immune serum as the negative control (supplementary material, Figures S1 and S2).

**IB and co-IP**

Antibodies used for IB experiments include anti-Dvl-2 (H-75; Santa Cruz, Dallas, TX, USA), anti-cyclin E (GeneTex, Hsinchu City, Taiwan), and anti-β-catenin (Cell Signaling Technology, Danvers, MA, USA). A goat anti-rabbit IgG (Jackson ImmunoResearch, West Grove, PA, USA) was used in conjunction with the polyclonal antibodies raised for the immune detection of the ASPM isoforms as described above. IB protein analysis was performed according to standard protocols. For co-IP, cells were lysed by non-denaturing lysis buffer (1 mm PMSF, 1 mm Na_3_VO_4_, 1 μg/ml pepstatin, 20 mm NaF, phosphatase inhibitor cocktail, 0.5% NP-40, and 10% glycerol in PBS) and the lysates (1 mg) were cleared by incubation with 50% protein A-Sepharose bead slurry, after which 1 ml of the cleared lysates was incubated with antibody-conjugated 50% protein A-Sepharose beads and 10 μl of 10% BSA overnight at 4°C. The beads were washed three times with washing buffer (0.5% NP-40, 0.1% Triton X-100, 1 mm PMSF, and 1 mm Na_3_VO_4_ in PBS). Proteins were revealed after SDS/PAGE and immunoblotting with the indicated antibodies.

**Gene expression manipulations**

The specific knockdown (KD) of the expression of *ASPM* transcript variant 1 (*ASPM*-v1; NCBI RefSeq: NM_018136.4; Figure 1A), which encodes ASPM-iI, by synthesizing several *in silico* predicted ASPM exon 18 (unique to *ASPM*-v1)-specific small hairpin RNA (shRNA) oligonucleotides using BLOCK-iT^TM^ RNAi Designer (Invitrogen). We constructed lentiviral vectors expressing each of these siRNAs by ligating the shRNA or microRNA sequence containing both sense and antisense strands, separated by a 9-bp loop region for directional cloning into pGLV2-U6-Puro (Biotools, Taipei, Taiwan). An shRNA oligonucleotide (clone #ASPM-vI-shRNA-4) which has a 21-nucleotide target sequence of 5'-GAGCTGCTATCACTTTACAGC-3' was selected for the subsequent development, due to its superior efficacy (data not shown). Nonspecific KD of both *ASPM*-vI and *ASPM* transcript variant 2 (*ASPM*-vII; NCBI RefSeq: NM_001206846), which encodes ASPM-iII, was achieved by lentivirus-mediated RNAi using a commercial shRNA oligonucleotide (clone TRCN0000118905) in the lentivector pLKO.1-puro (MISSION shRNA lentiviruses; Sigma-Aldrich, St Louis, MO, USA). A non-target control (SHC002V; Sigma-Aldrich) was used as the control. Multiple (two or three) rounds of lentiviral infections were carried out to achieve a satisfactory (> 80%) KD effect, as verified by RT-qPCR and immunoblotting analysis. Lentivirus was produced in Lenti-X 293T^TM^ cells (Clontech/Takara Bio, Mountain View, CA, USA) using the packaging vectors pMD2.G (Addgene #12259) and psPAX2 (Addgene #12260) to boost viral titer.

**Flow cytometry and tumorsphere assays**

Cells were dissociated, antibody-labeled (1–2 μg per 10^6^ cells × 1 h), and resuspended in HBSS/2% FBS. The ALDEFLUOR assay (StemCell Technologies, Vancouver, BC, Canada) was performed according to the manufacturer’s recommendation. Flow cytometry was done using a FACSCanto^TM^ II flow cytometer (BD Biosciences) with the electronic gating set according to cells stained with the corresponding isotype-matched control IgG. The tumorsphere assay was performed as previously described [20]. For limiting dilution assay, cells were plated in limiting dilution (200, 100, 50 cells per well) in 96-well plates in the respective culture media. The presence of tumorspheres was evaluated after 5 days. Experiments were performed in six replicates, and the results from three independent experiments were analyzed.

**Immunofluorescence (IF) and immunohistochemistry (IHC) analyses**

Cells grown on cultures plastics were fixed with 4% paraformaldehyde in PBS for 10 min at room temperature and then rinsed with 0.2% Triton X-100 in PBS. For paraffin-embedded sections, PDAC tissues were deparaffinized, hydrated, and immersed in citrate buffer at pH 6.0 for epitope retrieval in a microwave oven. Endogenous peroxidase activity was quenched in 3% hydrogen peroxide for 15 min, and slides were then incubated with 10% normal horse serum to block nonspecific immunoreactivity. The anti-ASPM-iI antibody (1:1600; described above) was subsequently applied and detected by using the DAKO EnVision kit (Dako, Carpinteria, CA, USA). Alexa Fluor 488- and Alexa Fluor 555-labeled secondary antibodies (Invitrogen, Carlsbad, CA, USA) were used at a 1:200 dilution and incubated for 1 h. Cells and slides were then mounted in ProLong Gold (Invitrogen). Confocal and IF imaging was performed using a Nikon Digital Eclipse C1 confocal microscope system and an Olympus IX81 fluorescence microscope, respectively. All IF and IHC staining was independently evaluated and verified by two expert pathologists (WYC and CTL) in a randomized manner. The staining intensities of markers were quantified at the single-cell level, with at least 300 tumor cells counted per tumor (three tissue sections per tumor; at least 100 tumor cells counted per section).

**Statistical analysis**

The statistical programming language R (https://cran.r-project.org) and SPSS 10.0 software (SPSS, Chicago, IL, USA) were used to conduct the statistical analysis of our data. Two-tailed Student’s *t*-test was used for simple significance testing for continuous data, and two-tailed chi-square tests were used for significance testing for categorical data. Spearman’s rank correlation was used for correlation analysis. Survival curves were generated using the Kaplan–Meier method. The curves were plotted and compared using the log-rank test using Prism 6.01 software (GraphPad Software, La Jolla, CA, USA). A cut-off value that best discriminates between groups with respect to outcome was determined using the concordance index, where an index of 1.0 is perfect discrimination [21]. The data from the limiting dilution assay were analyzed and plotted using ELDA software (http://bioinf.wehi.edu.au/software/elda/index.html). The likelihood ratio test and chi-square test were used to assess the significance.
