# Supplementary material for: The differential distributions of ASPM isoforms and their roles in Wnt signaling, cell cycle progression, and pancreatic cancer prognosis
Source: J Pathol. 2019 Oct 23;249(4):498–508. doi: 10.1002/path.5341 (PMC6899738; doi:10.1002/path.5341)
Supplement: Supplementary file 2 — Figure S1. The performance of isoform‐specific anti‐ASPM antibodies in PDAC cells Figure S2. ASPM‐iI and ASPM‐iII do not interact with each other in PDAC cells Figure S3. Knock‐down (KD) of ASPM transcripts or ASPM‐vI expression does not induce apoptosis in PDAC cells [file PATH-249-498-s001.docx]

**The differential distributions of ASPM isoforms and their roles in Wnt signaling, cell cycle progression, and pancreatic cancer prognosis**

Hsu C- C *et al. J Pathol* DOI: 10.1002/path.5341

**Supplementary Figures S1–S3**

**
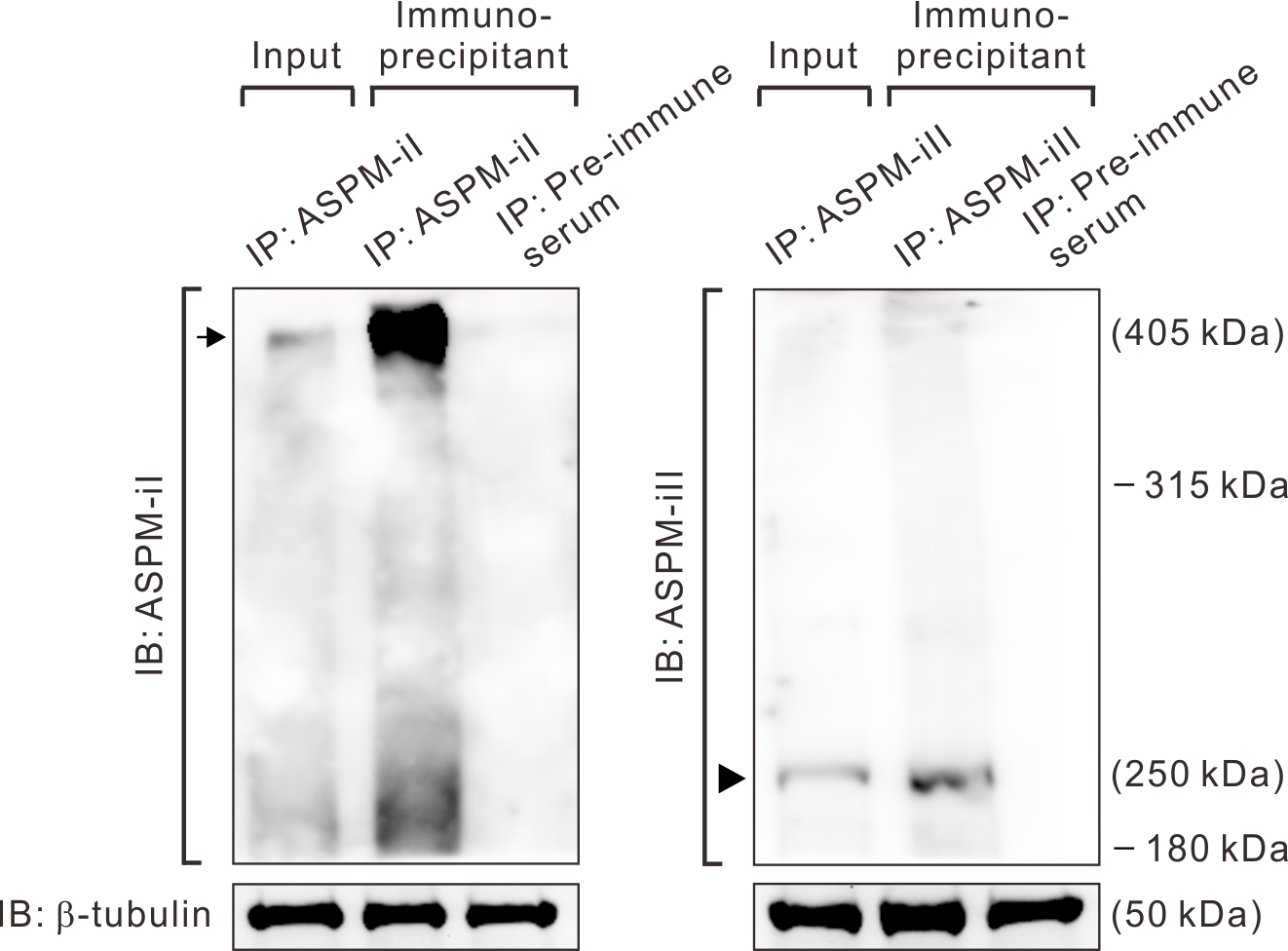
**

**Figure S1.** The performance of isoform-specific anti-ASPM antibodies in PDAC cells. The protein lysate of NCKUH-SP-1 cells was immunoprecipitated with anti-ASPM-iI (left) or anti-ASPM-iII antibody (right). The total lysate (input) or the immunoprecipitant was then blotted with the polyclonal anti-ASPM-iI (left) or the polyclonal anti-ASPM-iII antibody (right) to demonstrate the successful detection of the ASPM-iI (arrow; ~405 kDa) or the ASPM-iII protein (arrowhead; ~250 kDa) in the total lysate and the immunoprecipitant, respectively. The respective pre-immune serum was included in the blots to verify the specificity of the polyclonal antibodies. β-Tubulin was included as a loading control.


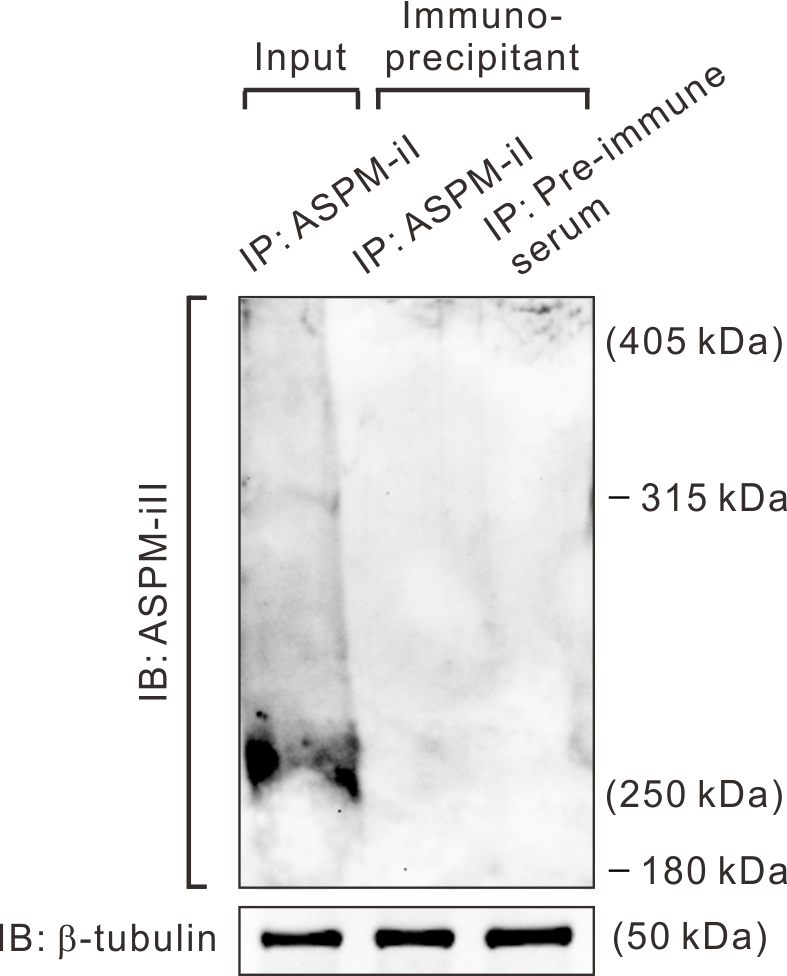


**Figure S2.** ASPM-iI and ASPM-iII do not interact with each other in PDAC cells. The protein lysate of NCKUH-SP-1 cells was immunoprecipitated with the polyclonal anti-ASPM-iI antibody. The total lysate (input) or the immunoprecipitant was then blotted with the anti-ASPM-iII antibody. Note that the ASPM-iII protein (arrowhead; ~250 kDa) was detected in the input but not in the anti-ASPM-iI immunoprecipitant. β-Tubulin was included as a loading control.


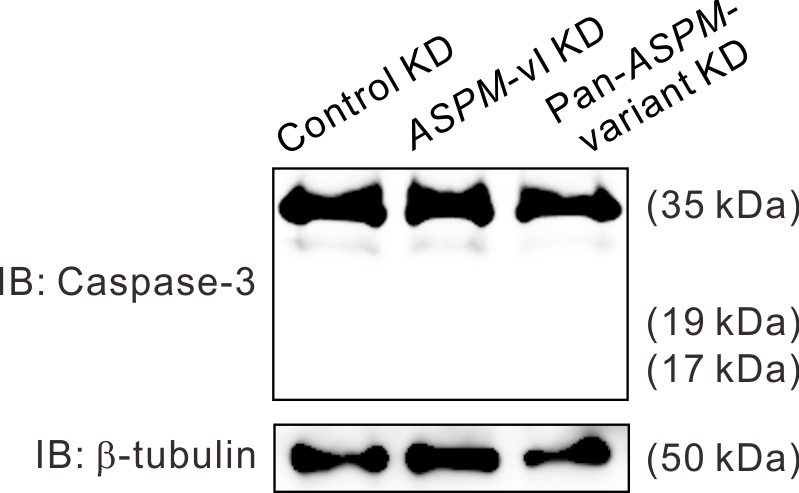


**Figure S3.** Knockdown (KD) of *ASPM* transcripts or *ASPM*-vI expression does not induce apoptosis in PDAC cells. Representative immunoblots of caspase-3 in the protein lysates of NCKUH-SP-1 cells lentivirally transduced with control shRNA, *ASPM*-vI-specific shRNA, or a pan-*ASPM*-variant shRNA are shown. Note that the cleavage products of caspase-3 (17 or 19 kDa) were not detectable in *ASPM*-vI KD or pan-*ASPM*-variant KD cells. β-Tubulin was included as a loading control.
