# Supplementary material for: The differential distributions of ASPM isoforms and their roles in Wnt signaling, cell cycle progression, and pancreatic cancer prognosis
Source: J Pathol. 2019 Oct 23;249(4):498–508. doi: 10.1002/path.5341 (PMC6899738; doi:10.1002/path.5341)
Supplement: Supplementary file 3 — Table S1. Clinical characteristics of the patients in the NCKUH cohort Table S2. Multivariate Cox regression model predicting overall survival by the ASPM‐iI Staining Index and clinico‐pathological criteria. [file PATH-249-498-s002.docx]

**The differential distributions of ASPM isoforms and their roles in Wnt signaling, cell cycle progression, and pancreatic cancer prognosis**

Hsu C-C *et al. J Pathol* DOI: 10.1002/path.5341

**Table S1.** Clinical characteristics of the patients in the NCKUH cohort

| **Variables** | **Number** |
| --- | --- |
| Number | 50 |
| Gender  Male  Female | 38 (76.0%)  12 (24.0%) |
| Age, years |  |
| Mean (SEM) | 62.2 (1.7) |
| Histology |  |
| Ductal adenocarcinoma  Pathological grade  1  2  3 | 50 (100%)  7 (14.0%)  28 (56.0%)  15 (30.0%) |
| TNM stage |  |
| T1 | 5 (10.0%) |
| T2 | 5 (10.0%) |
| T3  T4 | 36 (72.0%)  4 (8.0%) |
| N0 | 25 (50.0%) |
| N1 | 25 (50.0%) |
| Initial serum CA19-9 level, U/ml  Mean (SEM) | 447.8 (85.7) |
| Adjuvant chemotherapy  Survival status  Dead | 27 (54.0%)  36 (72.0%) |
| Alive | 14 (28.0%) |

SEM, standard error.

**Table S2.** Multivariate Cox regression model predicting overall survival by the ASPM-iI staining index and clinico-pathological criteria

|  | **Hazard ratio** | **95% CI** | ***P* value** |
| --- | --- | --- | --- |
| Age (per 10) | 0.296 | 0.809–1.455 | 0.586 |
| T stage (≥ 3 versus < 3) | 0.203 | 0.372–1.858 | 0.652 |
| N stage (1 versus 0) | 0.091 | 0.441–1.821 | 0.762 |
| CA-199 | 3.639 | 0.985–3.072 | 0.056 |
| ALDH-1 staining index (high versus*.* low) | 0.837 | 0.304–1.543 | 0.360 |
| ASPM-iI staining index (high versus low) | 5.863 | 1.338–15.896 | 0.015 |

CI, conference interval; ALDH, aldehyde dehydrogenase; ASPM-iI, ASPM isoform 1.
